# Ethische Zielkonflikte in der Corona-Krise

**DOI:** 10.1007/s41358-021-00267-2

**Published:** 2021-06-17

**Authors:** Klaus Steigleder

**Affiliations:** Fakultät für Philosophie und Erziehungswissenschaften, Arbeitsbereich Angewandte Ethik, Ruhr-Universität Bochum, Universitätsstraße 150, 44801 Bochum, Deutschland

Die gegenwärtige Corona-Pandemie und -Krise fordert die Ethik in vielfältiger Weise heraus. Die Pandemie stellt ein „Public Health“-Problem dar. Im Fokus stehen deshalb öffentliche und gesellschaftliche Aufgaben etwa der Eindämmung des Infektionsgeschehens. Diese dienen auch dem Schutz von Individuen, aber nicht einheitlich und in der gleichen Weise. Das öffentliche Interesse, die Interessen bestimmter Gruppen und die Interessen einzelner Personen können u. U. stark divergieren. Auch ist unklar, worin das öffentliche Interesse genauer besteht. Welche Aufgaben sind neben dem Infektionsschutz zu berücksichtigen und wie weit darf der Infektionsschutz gehen? Ein notorisches Problem ist die Bezugsgröße von „Public Health“-Problemen und -Aufgaben (national, international, regional oder global?) und den damit einhergehenden Interessenskonflikten und divergierenden (räumlichen und zeitlichen) Aufgabenbestimmungen (Faden et al. 2019). Schließlich konzentriert sich die Angewandte Ethik bislang vor allem auf abgrenzbare Bereiche und die für diese Bereiche charakteristischen Problemstellungen und stößt angesichts der komplexen bereichsübergreifenden Herausforderungen der Gegenwart, zu denen auch die Corona-Pandemie gehört, an ihre Grenzen. Nicht zufällig wird die die Pandemie betreffende ethische Diskussion in Deutschland von engumrissenen medizinethischen Überlegungen dominiert, die sich auf einen medizinisch verstandenen direkten Lebensschutz konzentrieren.

Ziel meines Essays kann es nicht sein, diese Probleme auf wenigen Seiten zu lösen. Stattdessen will ich einige problematische Engführungen herausstellen, auf weitreichende Fehleinschätzungen hinweisen und wichtige Aufgaben für die weitere Forschung benennen. Dabei werde ich in diesem Rahmen nicht auf die globalen Problemstellungen eingehen können.

## Bedeutung und Grenzen des Lebensschutzes

Rechte-basierte Moraltheorien gehen davon aus, dass die Menschen gleiche Rechte auf die notwendigen Bedingungen haben, ihr Leben führen zu können.^1^ Dazu gehören z. B. Freiheit, Leben, physische und psychische Integrität, Eigentum. Die *insgesamt* notwendigen Voraussetzungen dafür, sein Leben führen zu können, sind aber konkret in unterschiedlichem Maße unverzichtbar, von völlig unverzichtbar wie das Leben bis hin zu Voraussetzungen, deren Abwesenheit uns erheblich in unseren Möglichkeiten einschränkt, unser Leben zu führen, und schließlich Voraussetzungen, die in unterschiedlichem Maß situativ oder zeitweise verzichtbar sind. Entsprechend sind unsere moralischen Rechte nicht einfach alle gleichrangig, sondern sie bilden untereinander eine Rangordnung. Fallweise kann deshalb ein ranghöheres Recht der einen Person ein rangniedrigeres Recht einer anderen Person überwiegen. Wenn A zu verhungern droht und B Nahrungsmittel im Überfluss hat, dann sind A oder C unter Umständen dazu berechtigt, B etwas von seinen Nahrungsmitteln zugunsten von A wegzunehmen.

Ich werde weiter voraussetzen, dass die moralischen Rechte der Menschen nicht nur negative Rechte, also Rechte auf Unterlassung, sind, sondern (zugleich) auch positive Rechte, also Rechte auf Hilfeleistung. Wegen der grundsätzlichen Gleichheit der Rechte der Menschen, sind Rechte auf Hilfeleistung immer situativ bedingt. Eine Person hat immer dann einen Anspruch auf Hilfeleistung, wenn ein grundlegendes Recht von ihr bedroht ist, sie sich selbst nicht helfen kann und eine andere Person ohne vergleichbare Kosten zur Hilfeleistung in der Lage ist. Im Falle chronischer Hilfsbedürftigkeit würde die Hilfeleistung einzelne Personen überfordern, sie kann aber kollektiv organisiert und (etwa sozialstaatlich) institutionalisiert werden (Steigleder 2014). Für den Einzelnen besteht dann vor allem die Pflicht, die entsprechenden Institutionen zu unterstützen. Daraus erwächst wiederum ein Recht, im Falle von Bedürftigkeit die entsprechenden Institutionen in Anspruch nehmen zu können. Zu guter Letzt schließen die moralischen Rechte der Personen ein Recht auf den effektiven Schutz dieser Rechte ein. Auch dies macht staatliche Institutionen notwendig. Es bedarf aber auch internationaler und globaler Institutionen. Hier sind aber, sowohl was die theoretische Bestimmung als auch die praktische Umsetzung anbelangt, noch viele Fragen offen.

In der Rangordnung der moralischen Rechte nimmt das Leben einen besonderen Platz ein. Es ist völlig unverzichtbar dafür, sein Leben führen zu können. Es liegt deshalb nahe, von einem absoluten Recht auf Leben auszugehen, das durch keine anderen Rechte überwogen werden kann. Eine solche Vorstellung scheint die Politik in der Corona-Krise und die Abwägung von Zielkonflikten in Deutschland und vielen anderen Ländern zu leiten. Wir begegnen dieser Vorstellung in der Rede vom unbedingten Vorrang der Gesundheit oder des Gesundheitsschutzes. Die Vorstellung führt aber in die Irre.

Falls es ein absolutes Recht auf Leben gibt, dann ist es das Recht eines Unschuldigen, nicht zum Gegenstand einer intendierten Tötungshandlung gemacht zu werden (Gewirth 1981). Dieses kann aber für die Bestimmung angemessener Maßnahmen in der Corona-Krise nicht einschlägig sein. Die Ansteckung durch SARS-CoV 2 (im Folgenden spreche ich der Einfachheit halber vom Corona-Virus) ist keine direkte Tötungshandlung, geschweige denn eine intendierte. Vielmehr sind hier Risiken involviert: das Risiko, Träger des Corona-Virus zu sein, das Risiko, einen anderen mit dem Virus zu infizieren, das Risiko, dass eine solche Infektion bei einem Infizierten ggf. zu einer schweren Erkrankung führt, und das Risiko, an dieser Erkrankung zu versterben. Nach allem, was wir wissen, stellen sich diese Risiken je nach den Orten und Arten der Begegnung und je nach den möglichen Betroffenen höchst unterschiedlich dar. Die Risiken von Covid-19 sind real und nicht zu unterschätzen, aber sie sind offensichtlich begrenzt und (weitgehend) beherrschbar.^2^

Dass das Virus und der Umgang mit ihm Risiken und Risikoabwägungen involviert, erschwert die Entwicklung einer guten Politik im Umgang mit dem Virus ganz erheblich. Offensichtlich sind der bewusste Umgang mit Risiken und die Kompetenz für Risikoabwägungen unterentwickelt. In der Ethik stellen Risikobewertungen immer noch eine empfindliche Lücke dar.^3^

Ein absoluter Lebensschutz kann nicht Aufgabe staatlicher Institutionen sein. Letztlich geht es darum, die Rechte der Personen insgesamt zu sichern und im Rahmen der Möglichkeiten sicherzustellen, dass die Bürger über die notwendigen Voraussetzungen verfügen, ihr Leben führen zu können. Schon in Bezug auf den Schutz Unschuldiger, nicht zum Gegenstand intendierter Tötungshandlungen zu werden, sind die moralisch legitimen Möglichkeiten des Staates, dies zu verhindern, begrenzt. Dies gilt erst recht für die Vermeidung und Reduktion von Ansteckungsrisiken, die das Risiko schwerer und u. U. tödlicher Krankheitsverläufe mit sich bringen. Der Aufbau und der Erhalt eines funktionierenden Gesundheitswesens stellen mit Blick auf die Rechte der Menschen eine fundmentale staatliche Aufgabe dar, aber der Gesundheitsschutz ist weder absolut noch durchweg vorrangig.

Denn erstens gilt es den Schutz einer Vielzahl von Rechten sicherzustellen. Und in diesem Zusammenhang geht es auch darum, welche Lebenschancen Menschen haben. Hier ist es relevant, ob Menschen Fähigkeiten, die sie mit erheblichem Einsatz erworben oder entwickelt haben, nutzen können oder die Früchte dessen, was sie mühevoll aufgebaut haben, genießen können, ob also Fähigkeiten, Pläne und Lebensentwürfe entwickelt und verfolgt werden können.

Zweitens ist zu beachten, dass der Schutz von Gesundheit und Leben nicht auf den Schutz vor Ansteckung beschränkt ist. Um diesen Schutz zu gewährleisten, muss auch andere wichtige medizinische Versorgung in ausreichendem Maß angeboten werden. Durch das Verschieben von Arztbesuchen, Untersuchungen, Therapien oder Operationen wird diese Versorgung u. U. aber empfindlich beeinträchtigt. Relevant sind auch die gesundheitlichen Folgen mangelnder Bewegung, veränderten Ernährungsverhaltens, der längerfristigen Auswirkungen nicht realisierter Bildungschancen, massiver Eingriffe in individuelle Entwicklungsphasen, der Beschneidung von Karrierechancen, der Verhinderung von für das weitere Leben entscheidenden Begegnungen, psychischer Probleme und nicht zuletzt die Gefahr zunehmender Suizide derer, die angesichts der Corona-Präventionsmaßnahmen und deren erwartbaren Folgen jede Hoffnung verlieren.

Drittens dürfen die Grundlagen des institutionellen Schutzes von Rechten nicht riskiert werden. Das Kriterium der nicht vergleichbaren Kosten gilt auch für die Bestimmung der Aufgaben und Grenzen staatlichen Handelns. Um zunächst ein Beispiel aus einem anderen Bereich anzuführen: Wenn die Aufnahme von Flüchtlingen ab einem bestimmten Ausmaß die Funktionsfähigkeit staatlicher Institutionen oder den politischen Zusammenhalt in einem Staatswesen bedroht, dann ist die weitere Aufnahme von Flüchtlingen (vorerst) nicht nur nicht verpflichtend, sondern moralisch falsch. Wesentlich für den Schutz der Rechte der Bewohner eines Staates ist eine dauerhaft funktionierende Wirtschaft. Diese darf nicht gefährdet werden, weil eine dysfunktionale Wirtschaft einerseits den Betroffenen die Lebensgrundlage entzieht und weil sie andererseits zu einer dauerhaften Schwächung der Institutionen des Staates führt, die dem effektiven Schutz der Rechte der Menschen dienen, vom Gesundheitswesen über die sozialen Sicherungssysteme bis hin zum Bildungssystem.

## Die Problematik von Lockdowns angesichts der Corona-Pandemie

Die deutsche Politik (auf die ich mich im Folgenden der Einfachheit halber beziehe) der Lockdowns angesichts der Corona-Pandemie war und ist vor allem von drei Prämissen bestimmt: der Prämisse des Vorrangs des Lebensschutzes, der damit eng verbundenen Prämisse der Verhältnismäßigkeit der mit den Lockdowns verbundenen Einschränkung von Rechten und der Prämisse der Vertretbarkeit und Beherrschbarkeit der Folgerisiken der Lockdowns. Alle drei Prämissen sind hoch problematisch. Freilich ist nach den verfügbaren Handlungsalternativen zu fragen. Hier bestanden oder bestehen im Wesentlichen vier Alternativen: (1.) Die Zulassung einer unkontrollierten Ausbreitung des Virus, (2.) der Versuch, die größtmögliche Normalität aufrechtzuerhalten oder herzustellen, die mit dem gezielten Schutz von Risikopersonen vereinbar ist, (3.) ein konkret zielgerichteter und strikt limitierter Lockdown zur Schaffung der Voraussetzungen für die Umsetzung von Alternative (2.), (4.) ein pauschal zielgerichteter und nicht klar limitierter Lockdown.

In Deutschland wurde wiederholt die Alternative (4.) gewählt. Diese ist aber nicht zu rechtfertigen. Die beste Alternative ist wohl (2.), und Alternative (3.) ist dann gerechtfertigt, wenn aufgrund von bestehenden Beschränkungen, Fehlern oder Versäumnissen (2.) noch nicht oder nicht anders mehr realisiert werden kann. Die Rechtfertigung für (3.) ist also, dass sich das Virus nicht (mehr) ausreichend beherrschen und sich (2.) nicht anders (mehr) erreichen lässt.

Um die Probleme von Alternative (4.) aufzuzeigen, möchte ich näher herausstellen, wie Alternative (3.) aussehen würde. Dies kann am einfachsten durch einen Blick auf den ersten Lockdown im März 2020 geschehen. Ein Lockdown war unvermeidlich geworden, nachdem der rechtzeitige Beginn der Implementierung von Alternative (2.) verpasst worden war und es auch eine Reihe von Hindernissen gab, Alternative (2) wirksam zu implementieren: (a) Die Infektionen nahmen exponentiell zu, und man wusste nicht, wie man diese Zunahme verlässlich anders stoppen konnte. (b) Es fehlte an Schutzausrüstung und Gesichtsmasken. (c) Es fehlte ausreichendes handlungsrelevantes Wissen in Bezug auf das Virus. (d) Es fehlten ausreichende Testmöglichkeiten, die es bei rechter Anwendung erlauben, gezielte Schutz- oder Eindämmungsmaßnahmen zu ergreifen, ohne gleich ganze Einheiten (Fabriken, Schulen, Universitäten, Stadtteile, Städte etc.) lahmzulegen. Dies erfordert die Beschaffung der für die Tests erforderlichen Materialien, die Organisation und Koordination ausreichender Einrichtungen für die Durchführung der Tests, den Ausbau von Laborkapazitäten für die Auswertung der Tests (sieben Tage die Woche, rund um die Uhr), den Aufbau und Ausbau der Kommunikationsmöglichkeiten, um die Testergebnisse zu kommunizieren. (Noch heute, Stand Mai 2021, drucken Labore die auf dem Computerbildschirm angezeigten Ergebnisse von Corona-Tests aus, um sie dann mit Hilfe eines dazu eigens angeschafften Faxgerätes an das zuständige Gesundheitsamt zu übermitteln.) (e) Es fehlte eine personelle, zeitliche, räumliche und instrumentelle Aufstellung und Ausstattung der Gesundheitsämter, um Kontakte und mögliche Infektionswege an jedem Tag der Woche nachzuverfolgen und Quarantänen anzuordnen und zu organisieren, aber auch Befunde auszuwerten, Cluster und Probleme zu erkennen und darauf zu reagieren. (f) Es fehlten die technischen Möglichkeiten, Kontaktnachverfolgungen zu implementieren („Corona-App“). Zugleich fehlten Regelungen, den Datenschutz an die Erfordernisse der Notsituation anzupassen, und Anstrengungen der Politik, diese Erfordernisse zu erklären. (g) Es fehlten Konzepte, wie ein Schulbetrieb unter Corona-Bedingungen bestmöglich aufrechterhalten kann, und es bestanden eklatante Mängel in der Ausstattung der Schulen (sanitäre Anlagen, Internet, digitale Endgeräte etc.). (h) Es fehlten Konzepte für den öffentlichen Nahverkehr, wie dieser (ggf. unter Einbeziehung von Reisebusunternehmen) in Verbindung mit der organisatorischen Anpassung der Arbeitsorganisation und etwa des Schulunterrichts (versetzte Anfangszeiten, geringere Klassengrößen, Schichtunterricht etc.) so organisiert werden kann, dass er möglichst risikolos genutzt werden kann. (i) Es fehlten Konzepte, welche Umrüstungen und Maßnahmen erforderlich sind, um die Nutzung von Geschäften, Gaststätten, Sportmöglichkeiten, Bibliotheken, Museen, Theatern, Konzertsälen etc. zu ermöglichen. (j) Es fehlten Kriterien, welche Aktivitäten bis auf Weiteres nicht oder nur sehr eingeschränkt und im Verbund mit hohen Sicherheitsmaßnahmen möglich sind.

Ein Lockdown der Art (3.) versucht diese Probleme soweit und so schnell wie möglich zu überwinden. Das heißt, dass er, in Abwägung mit anderen Zielgrößen, von vornherein eine Tiefe haben muss, dass das Infektionsgeschehen wirksam gestoppt und reduziert werden kann. Zugleich müssen von *Tag 1* des Lockdowns an auf allen Ebenen Pläne erarbeitet werden und die notwendigen Maßnahmen definiert und getätigt werden, um den Lockdown in unterschiedlichen betroffenen Bereichen schnellstmöglich wieder zu beenden. Selbstverständlich muss nicht abgewartet werden, bis alle aufgeführten Mängel vollständig beseitigt sind. Sie müssen zunächst einmal so weit angegangen werden, dass der Lockdown möglichst bald gelockert werden kann. Die Implementation von Strategie (2.) muss schrittweise und kontrolliert erfolgen.

Der Unterschied zwischen Alternative (3.) und der tatsächlich verfolgten Alternative (4.) lässt sich exemplarisch daran ersehen, dass in Deutschland die Kultusministerkonferenz in der siebten Woche des ersten Lockdowns beschlossen hat, dass Hygienekonzepte für eine Wiederaufnahme des Schulbetriebs erarbeitet werden müssen. Die meisten der benannten Erfordernisse sind bis heute nicht ausreichend gelöst oder überhaupt erst angegangen. Es ist eine wichtige Aufgabe zu erkennen, warum das so ist. Ich vermute, dass eine Antwort darin besteht, dass die Politik in einer Marktwirtschaft daran gewohnt ist, dass viele Dinge sich durch unternehmerische Initiative regeln und die Aufgabe von Politik sich hier weitgehend darauf beschränkt, diese rechtlich zu flankieren oder zu steuern. Es wird nicht gesehen, dass die Corona-Krise auf diese Weise nicht in der erforderlichen Zeit bewältigt werden kann. Der Orientierungspunkt müsste stattdessen eine Art Kriegswirtschaft sein, in der es viele Dinge von oben nach unten planmäßig anzugehen und zu organisieren gilt. Weniger martialisch lässt sich auch von der Notwendigkeit einer vorübergehenden *Herrschaft des Plans* sprechen. Eine weitere Antwort ist, dass ausgedehnte, eingespielte Bürokratien offensichtlich die Initiative von Macherinnen und Machern nicht begünstigen.

Alternative (1.) würde zu vielen durch die Implementation von Alternative (2.) vermeidbaren Todesfällen führen und ist schon deshalb nicht vertretbar. Zudem besteht die Gefahr des Verlusts der Funktionsfähigkeit von Wirtschaft und Gesellschaft. Ob und wie sich Alternative (2.) tatsächlich implementieren lässt, hängt an einem komplexen Zusammenspiel von unvermeidlich begrenztem „Tatsachen“wissen und normativen Wertungen. Die Entwicklung von Alternative (2.) und die Schaffung der Voraussetzungen dafür, sie dauerhaft aufrechtzuerhalten, ist die eigentliche Aufgabe. Sie anzugehen und zu bewältigen, daran wird ohnehin kein Weg vorbeiführen.

Die Impfstrategie in Deutschland folgte bislang (Stand Mai 2021) vor allem medizinethischen Kriterien des Lebensschutzes. Aus ethischer Sicht ist dies problematisch. Man hätte nicht nur nach den Risikogruppen fragen müssen, sondern auch nach den unterschiedlichen Schutzmöglichkeiten für diese Gruppen und nach der Relevanz des Impfschutzes bestimmter Gruppen für die Umsetzung von Alternative (2.). Die Impfung der Bewohner von Alters- und Pflegeheimen stellt für diese wohl den besten Schutz dar (vor allem wenn es nicht gelingt, die Übertragung des Virus durch Ärzte oder Pflegepersonal zu verhindern). Daraus lässt sich, wenn man ernst nimmt, dass es auch weitere Zielgrößen zu berücksichtigen gilt, noch nicht eine Impfung aller weitgehend kontaktarmen Achtzig- und Neunzigjährigen ableiten. Eine nähere Bearbeitung dieser Fragen ist wichtig. Dringlich ist es auch, die Aufgaben zu bestimmen, wie die Corona-Krise in globaler Perspektive angegangen werden muss.

## Footnotes

1 Siehe ausführlicher Steigleder (2017), Gewirth (1978); siehe auch Shue (2020).

2 Rabadan (2020); Christakis (2020); Horton (2021).

3 Hayenhjelm und Wolff (2012); Hansson (2013); Steigleder (2016, 2018).
